# The Regime Shift Associated with the 2004–2008 US Housing Market Bubble

**DOI:** 10.1371/journal.pone.0162140

**Published:** 2016-09-01

**Authors:** James Tan, Siew Ann Cheong

**Affiliations:** 1 Interdisciplinary Graduate School, Nanyang Technological University, Singapore, Republic of Singapore; 2 Division of Physics and Applied Physics, School of Physical and Mathematical Sciences, Nanyang Technological University, Singapore, Republic of Singapore; 3 Complexity Institute, Nanyang Technological University, Singapore, Republic of Singapore; East China University of Science and Technology, CHINA

## Abstract

The Subprime Bubble preceding the Subprime Crisis of 2008 was fueled by risky lending practices, manifesting in the form of a large abrupt increase in the proportion of subprime mortgages issued in the US. This event also coincided with critical slowing down signals associated with instability, which served as evidence of a regime shift or phase transition in the US housing market. Here, we show that the US housing market underwent a regime shift between alternate stable states consistent with the observed critical slowing down signals. We modeled this regime shift on a universal transition path and validated the model by estimating when the bubble burst. Additionally, this model reveals loose monetary policy to be a plausible cause of the phase transition, implying that the bubble might have been deflatable by a timely tightening of monetary policy.

## Introduction

The US housing market was the epicenter of the economic turmoil that roiled global financial markets in the past decade. This crisis had its roots in risky lending practices in the form of subprime mortgages given to individuals with poor credit ratings. When these borrowers could not repay their loans, the subprime mortgages soured and lenders failed, leading to a financial crisis in the US housing market known as the Subprime Crisis and culminating in the Global Financial Crisis [1–3]. On hindsight, one wonders why regulators allowed the housing bubble to grow out of proportions, and not nib it in the bud. It turned out that there are few sure signs of an asset bubble that people can agree on. A few more compelling detection of housing bubbles included Zhou et al., who used a faster-than-exponential growth and log-periodic oscillations in price indices to indicate the presence of housing bubbles [4]. Ohnishi et al. showed that land price distribution in Japan followed a log-normal distribution before and after the housing bubble and a power law distribution during the housing bubble [5]. More recently, Meng et al. studied the eigenvalues of correlation matrices of housing price indices across US states [6] and used the evolution of these eigenvalues to identify regime shifts in the US housing market.

**Fig 1:**
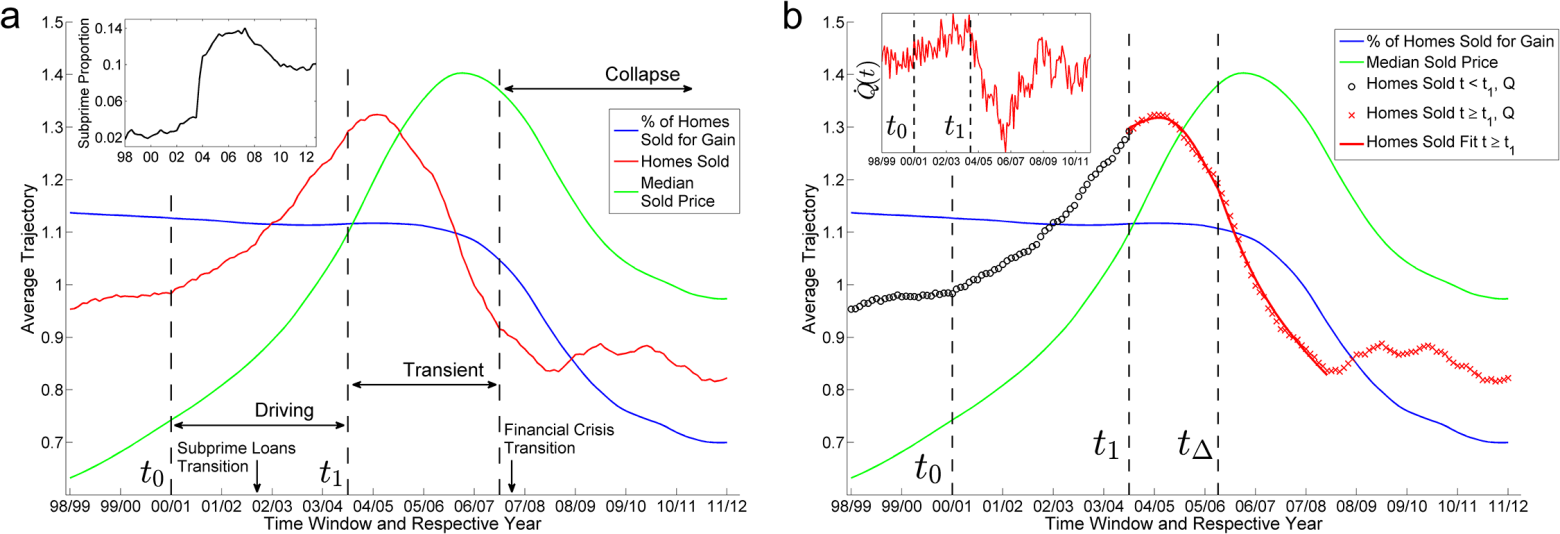
**(a) The average normalized trajectories of the three variables are plotted against time**. Dashed lines demarcate distinct periods of the trajectory of the US housing market. The start *t*_0_ and end *t*_1_ of the driving phase are marked by kinks in the homes sold trajectory and its gradient respectively while the start of the collapse phase is marked by a collapse in the median sale price and % of homes sold for gain trajectories. (inset) The line plot shows the subprime proportion of mortgages issued in the United States. The sudden jump in the fourth quarter of 2003 is marked on the main figure as the Subprime Loans Transition (October 2003). **(b) The average normalized trajectory of the percentage of homes sold for gain variable and the median sold price variable are plotted with the fits of the model to the homes sold trajectory, *Q*(*t*)**. The fitted parameter *t*_Δ_, which marks the start of a decline in the fundamental transaction volume, coincides with the start of a collapse in the median sold price variable and the percentage of homes sold for gain variable. (inset) The time derivative of the homes sold trajectory where a kink is seen at *t*_0_ that results in a gradual increase in $\dot{Q}\left(t\right)$ and where a kink is seen at *t*_1_ that results in a steep decline of $\dot{Q}\left(t\right)$.

Broadly speaking, the sequence of events in the US housing market can be separated into two time periods following: (1) an abrupt increase in the proportion of subprime mortgages out of all mortgages issued and, (2) the failure of large financial institutions from the default of subprime mortgages. We shall refer to the former as the Subprime Loans Transition and the latter event as the Global Financial Crisis Transition or the Financial Crisis Transition. The Subprime Loans Transition occurred in the fourth quarter of 2003 (Fig 1 inset) while the Global Financial Crisis Transition occurred in September 2008 with the failure of large financial institutions like Lehman Brothers and Merril Lynch. The Global Financial Crisis Transition marks the onset of the Global Financial Crisis when problems in the US housing market constituting the Subprime Crisis spilled over to the financial world at large. These transitions mark the beginning and the end of an excessively exuberant period in the US housing market.

Evidence supporting our assessment that the Subprime Loans Transition and the Global Financial Crisis Transition are discontinuous phase transitions comes in the form of statistical early warning signals prior to the transitions [7]. These signals work on the premise of critical slowing down, where the return rate to a stable equilibrium point decreases as the equilibrium point becomes less stable approaching a bifurcation point [8, 9]. When the system escapes the equilibrium point either through noise or going past the bifurcation itself, a phase transition will have taken place. Statistical signals arising from critical slowing down have been used as early-warning signals to discontinuous phase transitions for a wide variety of systems [10–17]. By using critical slowing down signals to identify regimes, we are able to detect instability where more traditional methods like the Markov regime switching model are less able to. In this paper, we are interested in understanding how the velocity of the transition changes as we cross the bifurcation point, where the energy barrier disappears. Additionally, the transition that we have observed occurs on a relatively long timescale. Therefore, we cannot model the data with a Markov regime switching model without introducing many more hidden states and overfitting the data in the process.

In our previous work, we have shown that critical slowing down signatures in the form of spectral reddening can be detected before the two transitions in two out of three Zillow.com variables associated with the US housing market [7]. The two variables are the number of homes sold and the percentage of homes sold for gain. The third variable is the median sold price. Here, we show that three additional critical slowing down signatures for the homes sold variable can also be detected. These three signatures are the autocorrelation, the skewness, and the variance. We also show that in the homes sold variable, the regime shift associated with the Subprime Loans Transition can be modeled as a transition on a bifurcating free energy landscape.

## Average Normalized Trajectory of the US Housing Market

In order to model the regime shift, we first construct the average normalized trajectories for each of the three variables: homes sold, median sold price, and percentage of homes sold for gain (Section S3 in S1 File). The average normalized trajectory is a time series obtained through a process of normalizing the data of every city and averaging this normalized data over all cities. We find that the homes sold variable *Q*(*t*) from the average normalized trajectory (Fig 1a) between *t*_0_ ≤ *t* ≤ *t*_*f*_ (where *t*_*f*_ = is the time window ending in May 2009) closely resembles $\dot{x}\left(t\right)$ of a discontinuous phase transition in the order parameter *x*(*t*). The transition can be described by the dynamical equation

$$\begin{array}{c} \frac{dx}{dt}=-\frac{\partial U(x,t)}{\partial x}. \end{array}$$ (1)

Here, *U*(*x*, *t*) is the coarse-grained free energy surface initially containing a double well potential. A saddle node bifurcation occurs during the driving phase, where *U*(*x*, *t*) is changing continuously. The saddle node bifurcation results in the annihilation of the initial potential well that the system was residing in, leading to a regime shift to the alternate potential well. *U*(*x*, *t*) may be approximated by a Taylor expansion. If the separation between the two regimes is small, we may expand up to the fourth order polynomial since it is the lowest order able to account for a transition between two alternate stable states. Therefore, *U*(*x*, *t*) may be written as

$$\begin{array}{c} U\left(x,t\right)=-A\left(t\right)x+B\left(t\right)x^{2}+C\left(t\right)x^{3}+D\left(t\right)x^{4}. \end{array}$$ (2)

Depending on the values of the parameters *A*(*t*), *B*(*t*), *C*(*t*), and *D*(*t*), the free energy *U*(*x*, *t*) will be a double well potential (i.e. two stable equilibrium points) if the roots of −∂*U*(*x*, *t*)/∂*x* are real and *D*(*t*) > 0. If the driving is turned on and off suddenly, then the start and end of the driving phase will be marked by kinks which might be observable in the data. The kinks observed at *t*_0_ and *t*_1_ (inset of Fig 1b) suggests that the driving started at *t*_0_ and stopped at *t*_1_. Hence, all four parameters *A*(*t*), *B*(*t*), *C*(*t*), and *D*(*t*) may be time-dependent during the driving phase, but become constants after *t*_1_ i.e. *A*(*t* ≥ *t*_1_) = *A*_1_, *B*(*t* ≥ *t*_1_) = *B*_1_, *C*(*t* ≥ *t*_1_) = *C*_1_ and *D*(*t* ≥ *t*_1_) = *D*_1_. This allows us to fix and estimate the parameters of *U*(*x*, *t*) after the driving phase, by fitting the model to data. In the Supporting Information, we also demonstrate this free energy model on simulated data from a regime shift in a one-dimensional grazing model from ecology (Section S5 in S1 File). Unfortunately, the proper forms of *A*(*t*), *B*(*t*), *C*(*t*) and *D*(*t*) during the driving phase cannot be determined from the data without additional mechanistic insights, because of the problem of overfitting (Section S6 in S1 File). We relate the homes sold variable *Q*(*t*) to *x*(*t*) by

$$\begin{array}{c} Q\left(t\right)=\frac{dx}{dt}+Q_{0}+Q_{g}t+Q_{\Delta }\left(t\right). \end{array}$$ (3)

In this equation, *Q*_0_ is the constant fundamental part of *Q*(*t*) expected from US demographics, while *Q*_*g*_ *t* accounts for the natural growth in homes sold due to growth in the US population. We estimate *Q*_*g*_ and *Q*_0_ with a linear regression (Fig C in S1 File). We also introduce *Q*_Δ_(*t*) to account for the observed decrease in *Q*(*t*) before and after the transition. We assume that this decrease is linear,

$$\begin{array}{c} Q_{\Delta }\left(t\right)=\left\{\begin{array}{cc} 0, & \;\text{for}\;t<t_{\Delta };\\ \frac{Q_{\text{end}}-Q_{0}}{t_{f}-t_{\Delta }}\left(t-t_{\Delta }\right), & \;\text{for}\;t_{\Delta }\leq t\leq t_{f}, \end{array}\right. \end{array}$$ (4)

where *t*_Δ_ is the time when *Q*_Δ_(*t*) starts decreasing from zero, and *Q*_end_ = *Q*(*t*_*f*_). In the Supporting Information, we look at other functional forms for *Q*_Δ_(*t*) and find that they do not alter the conclusions of this paper (Section S7 in S1 File).

## Results and Discussion

**Fig 2:**
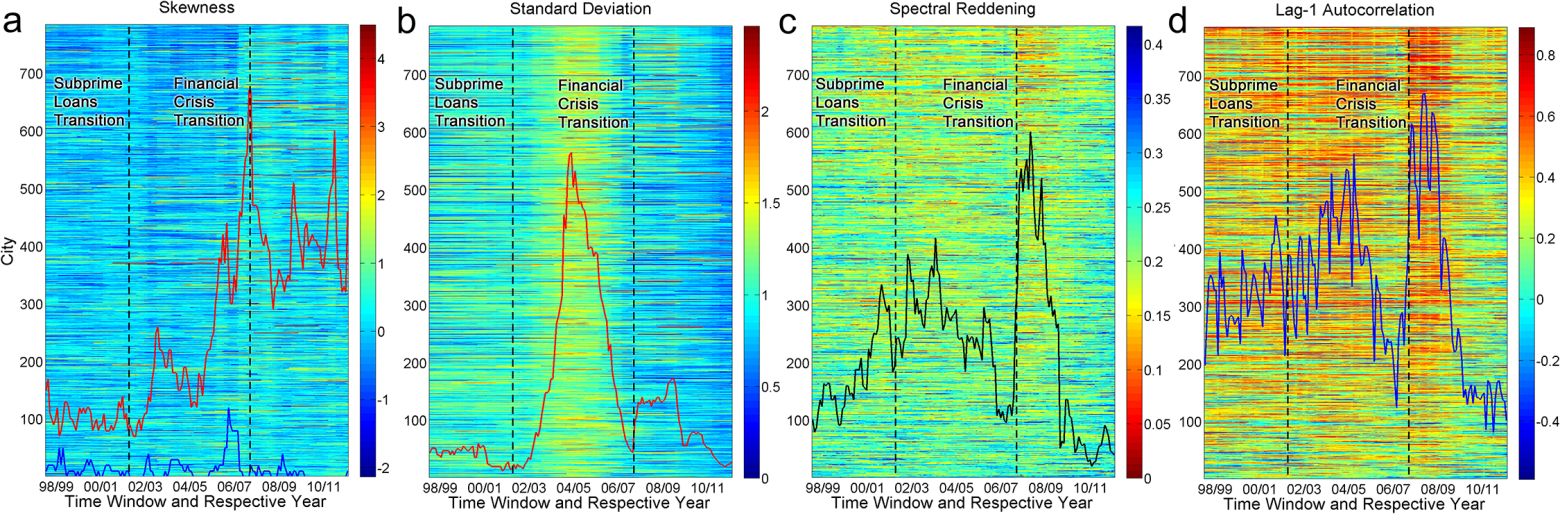
The vertical axis is the city index, ordered by the total number of transactions over the complete time series of the city. The horizontal axis is the time window and the color represents the strength of the corresponding early warning signal. Line plots indicate the number of cities that fall below a certain threshold for the corresponding early warning signal, scaled by an appropriate factor to fit onto the heat map. These line plots represent aggregated early warning signals that can be used to infer instability in the US housing market (Section S2 in S1 File). The first dashed line marks the time window with latest date corresponding to the Subprime Loans Transition whereas the second dashed line marks the time window with latest date corresponding to the Financial Crisis Transition. (a) Skewness. The blue line and red line corresponds to positive skewness, and negative skewness respectively. (b) Standard deviation. (c) Median frequency, *ω*_1/2_, a measure of spectral reddening. (d) Lag-1 autocorrelation.

Early warning signals are calculated for a set of time windows that are obtained by partitioning time series into overlapping two-year segments (time windows) that can be slid one month at a time (Section S2 of S1 File). Fig 2 shows the two periods of increasing early warning signals in the homes sold variable. The four signals that we computed are the skewness, standard deviation, spectral reddening and lag-1 autocorrelation. The skewness measures the asymmetry of a time series, the standard deviation measures the magnitude of fluctuations of a time series, and the spectral reddening and lag-1 autocorrelation are signals associated with the memory of a time series. These four signals are early warning signals that can appear prior to a regime shift [8]. They are based on the premise that exponential decay rates to perturbations of the system from a stable equilibrium point become slower with increasing instability as a bifurcation point is approached. Since the velocities of decays to the perturbations of the state variable also decay exponentially with the same decay rate as the state variable, then these signals can also be observed in the homes sold variable which we have identified as the velocity of the transition between regimes. We aggregated each type of signal across all cities to obtain four aggregated early warning signals corresponding to the four types of signals we are measuring. All aggregated signals show a statistically significant trend during the first period (Table A in S1 File). This first period is attributed to a large historic rise in the subprime proportion of mortgages issued in the US (the Subprime Loans Transition, which took place in the fourth quarter of 2003). However, only spectral reddening shows a significant trend before October 2003. In principle, as the energy well destabilizes to become shallower and asymmetric, all four early warning signals should grow in tandem. Indeed, spectral reddening, skewness, standard deviation, and autocorrelation all continued growing past the Subprime Loans Transition, but only the growth in spectral reddening is statistically significant before Oct 2003. This is likely due to the use of relatively long two-year time windows in computing the early warning signals. The Global Financial Crisis Transition marks the onset of the second period, which is when large financial institutions like Lehman Brothers and Merrill Lynch failed and when bank runs occurred in September 2008. All signals rose sharply and were statistically significant around this time (Table A in S1 File). Except for the standard deviation, these rising trends were statistically significant before September 2008. In the Supporting Information, we also show that the statistically significant trends in aggregated early warning signal around the time of both transitions are robust to time window length and detrending bandwidths (Section S8 in S1 File).

The average normalized trajectory of the homes sold variable (Fig 1a) from the 2000/2001 to 2007/2008 time window closely resembles the velocity $\dot{x}\left(t\right)$ of a one-dimensional system *x*(*t*) moving between two alternate fixed points on a bifurcating free energy surface. We model the phase transition using Eqs (1) and (2). Our assumption that the fixed points are close to each other before the start of the driving phase in Dec 2001 is justified, in view of deregulatory policies introduced in the late 1990s that inadvertently helped promote risk taking by lenders, and ultimately leading to the Subprime Crisis [18–22]. An example of such a policy is the repeal of the Glass-Steagall Act in 1999. These policies destabilize the stable fixed point, resulting in a shorter distance between the two regimes.

This free energy model allows us to explain in a unified way features seen in the average normalized trajectory, and the statistical early warning signals detected prior to the Subprime Loans Transition and the Global Financial Crisis Transition. According to the model, the kink observed in the homes sold variable at the start of the driving phase is due to the sudden changes in the time derivatives of changes in *A*(*t*), *B*(*t*), *C*(*t*) and *D*(*t*). As these parameters change, the initial stable fixed point is destabilized, and we approach a saddle-node bifurcation that corresponds to the Subprime Loans Transition. The destabilization leads to the observed early warning signals around the Subprime Loans transition. The positive skewness indicates that the bifurcation is approached from the positive direction which agrees with this interpretation. We expect this bifurcation to manifest itself across multiple variables of the housing market. However, the sharp increase in the proportion of subprime mortgages issued in the span of a few months suggests that this variable operates on a faster timescale than the transaction volume. The Global Financial Crisis Transition marked the end of the transition of the US housing market to an alternate fixed point. As suggested by the early warning signals, this new regime is only weakly stable. The positive skewness further indicates that it is weakly stable in the positive direction. However, the use of *U*(*x*, *t*) is still applicable since we are not modeling any movement past this alternate fixed point. No further transition out of this alternate stable state was observed in the homes sold variable, but both the percentage of homes sold for gain and the median sold price collapsed after the Global Financial Crisis Transition.

By fitting the model (Eqs 1–4) to the relevant portion of the homes sold trajectory *Q*(*t*_1_ ≤ *t* ≤ *t*_*f*_) (Fig 1b), we obtained the fit reported in Table B in S1 File. In the best-fit obtained, only a single well exists after the driving phase, which rules out the possibility that *Q*(*t*) could be explained without any driving in the model (Fig D in S1 File). The fitted *t*_Δ_ corresponds to the time window ending in March 2007. *t*_Δ_ marks the start of a fundamental decline, *Q*_Δ_(*t*), in the homes sold trajectory. Intuitively, the start of such a decline should coincide with the start of collapse in transaction price and the % of homes sold for gain. This is because a decline in home prices will inevitably lead to a decline in the % of homes sold for gain. It follows from loss aversion [23], a concept well known in economic theory, that a fall in transaction volume will occur when home owners are unwilling to transact for a loss. Thus, we expect the fundamental transaction volume, $Q\left(t\right)-\dot{x}\left(t\right)$, to decline in the collapse phase. Indeed, our fit confirms our expectation that *t*_Δ_ lies close to the start of collapse of both the median sold price and the homes solid for gain variables, where a first decrease in both trajectories is seen (Fig 1b).

Interestingly, the timings of the start and end of the driving phase suggest that the driving might be caused by the US federal funds rate. To see this, let us consider the timeline of the changes made to the Federal Reserve rate. The start of the driving, *t*_0_, occurred at the time window ending Dec 2001. In the same month, the federal funds rate had also decreased to 1.75%, the lowest in many decades. Thereafter, the federal funds rate was lowered two more times, in November 2002 and in June 2003. The federal funds rate started rising in June 2004, peaking in June 2006. Hence, if it is indeed true that low interest rates drove the US housing market up, then when the interest rates were raised between June 2004 and June 2006, the driving phase should stop, i.e. *t*_1_ should fall between June 2004 and June 2006. This was indeed the case as *t*_1_ corresponds approximately to the time window ending in June 2005, when the federal funds rate was at 3%. Moreover, this association between driving (causing destabilization) in *U*(*x*, *t*) and the federal funds rate makes economic sense because lower interest rates encourage risky lending practices which is now believed to have fueled the subprime mortgage crisis [24–28]. Thus, in this interpretation, a low federal funds rate at *t*_0_ encouraged risky lending activity, which became a driving force that destabilized the system, whereas a high federal funds rate dampened risky lending activity, leading to the end of the driving phase at *t*_1_. Additionally, the directions of the kinks at *t*_0_ and *t*_1_ are also consistent with such an interpretation since an increase in risky lending activity is expected to lead to an increase in transaction volume at *t*_0_ while a decrease in risky lending activity is expected to lead to a decrease in transaction volume at *t*_1_. The fact that the driving appears and disappears at different interest rates (1.75% and 3%) suggests a hysteresis effect. Within this energy landscape picture, the US housing market can be in two alternate stable states, healthy and collapsed, and can be driven from the healthy state to a collapsed state through a saddle-node bifurcation. Driving started when the federal funds rate was lowered to 1.75%, and stopped when the interest rate was increased to 3%. This transient driving phase can be interpreted as a housing bubble.

Finally, let us discuss the implications of the energy landscape modeling done in this paper. As proposed by many economists before us, government policies contributing to the Subprime Crisis include deregulation of the financial sector, encouragement of home ownership, and an extended period of low interest rates [19, 28, 29]. These three policy decisions were not directly influenced by each other, but together they created a conducive environment for the US housing bubble of 2004 to 2008. Of the three policies, financial deregulation and home ownership were motivated by the desire to create public goods. Monetary policy, on the other hand, is a value-neutral tool aimed merely at guaranteeing liquidity. If we suppose that the Federal Reserve could freely set their funds rate without encountering strong public opposition, would it have been possible in principle to avert the Subprime Crisis? In this paper, only the forcing-free phase of the average normalized homes sold variable can be robustly fitted to conclude that by the end of the driving phase, the basin of attraction for the normal regime of the US housing market had disappeared. However, the energy landscape model cannot be robustly fitted to the driving phase of the data due to the problem of overfitting. Regardless, for whatever initial energy landscape with two alternate stable states, a market collapse can be avoided if we end the driving phase sufficiently far from the bifurcation point. This gives us confidence that soft landings are possible for a wide range of energy landscapes and forcings.

## Supporting Information

S1 File Supporting Information. Supporting information pdf file containing additional information on data and methodology. This is inclusive of figures, tables and text.
(PDF)

## References

[1] Keys BJ, Mukherjee T, Seru A, Vig V. Did securitization lead to lax screening? Evidence from subprime loans. Q J Econ. 2010;125(1):307–362. 10.1162/qjec.2010.125.1.307

[2] Mian A, Sufi A. The consequences of mortgage credit expansion: Evidence from the U.S. mortgage default crisis. Q J Econ. 2009;124(4):1449–1496. 10.1162/qjec.2009.124.4.1449

[3] Demyanyk Y, Hemert OV. Understanding the Subprime Mortgage Crisis. Rev Financ Stud. 2011;24(6):1848–1880. 10.1093/rfs/hhp033

[4] Zhou WX, Sornette D. Is there a Real-Estate Bubble in the US? Physica A. 2006;361(1):297–308. 10.1016/j.physa.2005.06.098

[5] Ohnishi T, Mizuno T, Shimizu C, Watanabe T. Power Laws in Real Estate Prices during Bubble Periods. Int J Mod Phys Conf Ser. 2012;16:61–81. 10.1142/S2010194512007787

[6] Meng H, Xie WJ, Jiang ZQ, Podobnik B, Zhou WX, Stanley HE. Systemic Risk and Spatiotemporal Dynamics of the US Housing Market. Sci Rep. 2014;4(3655):1–7. 10.1038/srep03655 PMC3888986 24413626

[7] Tan JPL, Cheong SA. Critical Slowing Down Associated with Regime Shifts in the US Housing Market. Eur Phys J B. 2014;87(38):1–10.

[8] Scheffer M, Bascompte J, Brock WA, Brovkin V, Carpenter SR, Dakos V, et al Early-Warning Signals for Critical Transitions. Nature. 2009;461(7260):53–59. 10.1038/nature08227 19727193

[9] Veraart AJ, Faassen EJ, Dakos V, van Nes EH, Lü M, Scheffer M. Recovery Rates Reflect Distance to a Tipping Point in a Living System. Nature. 2012;481(7381):357–359. 10.1038/nature10723 22198671

[10] Boulton CA, Allison LC, Lenton TM. Early Warning Signals of Atlantic Meridional Overturning Circulation Collapse in a Fully Coupled Climate Model. Nat Commun. 2014;5(5752):1–9. 10.1038/ncomms6752 PMC4268699 25482065

[11] Krkošek M, Drake JM. On Signals of Phase Transitions in Salmon Population Dynamics. Proc R Soc B. 2014;281(1784):20133221 10.1098/rspb.2013.3221 24759855 PMC4043080

[12] van de Leemput IA, Wichers M, Cramer AOJ, Borsboom D, Tuerlinckx F, Kuppens P, et al Critical Slowing Down as Early Warning for the Onset and Termination of Depression. Proc Natl Acad Sci USA. 2014;111(1):87–92. 10.1073/pnas.1312114110 24324144 PMC3890822

[13] Meisel C, Kuehn C. Scaling Effects and Spatio-Temporal Multilevel Dynamics in Epileptic Seizures. PLOS ONE. 2012;7(2):1–11. 10.1371/journal.pone.0030371 PMC3281841 22363431

[14] Carpenter SR, Cole JJ, Pace ML, Batt R, Brock WA, Cline T, et al Early Warnings of Regime Shifts: A Whole-Ecosystem Experiment. Science. 2011;332(6033):1079–1082. 10.1126/science.1203672 21527677

[15] Held H, Kleinen T. Detection of Climate System Bifurcations by Degenerate Fingerprinting. Geophys Res Lett. 2004;31(23):L23207 10.1029/2004GL020972

[16] Wang R, Dearing JA, Langdon PG. Flickering Gives Early Warning Signals of a Critical Transition to a Eutrophic Lake State. Nature. 2012;492(7429):419–422. 10.1038/nature11655 23160492

[17] Guttal V, Jayaprakash C. Changing skewness: An early signal of regime shifts in ecosystems. Ecol Lett. 2008;11(5):450–460. 10.1111/j.1461-0248.2008.01160.x 18279354

[18] Xu YL. Does mortgage deregulation increase foreclosures? Evidence from Cleveland. Reg Sci Urban Econ. 2014;46:126–139. 10.1016/j.regsciurbeco.2014.03.003

[19] McCoy PA, Pavlov AD, Wachter SM. Systemic Risk through Securitization: The Result of Deregulation and Regulatory Failure. Conn L Rev. 2009;41:493.

[20] Immergluck D. Core of the Crisis: Deregulation, the Global Savings Glut and Financial Innovation in the Subprime Debacle. City Community. 2009;8(3):341–345. 10.1111/j.1540-6040.2009.01292_1.x

[21] Mishkin FS. Over the Cliff: From the Subprime to the Global Financial Crisis. J Econ Perspect. 2011;25(1):49–70. 10.1257/jep.25.1.49

[22] Wray LR. Lessons from the subprime meltdown. Challenge. 2008;51(2):40–68. 10.2753/0577-5132510205

[23] Kahneman D, Tversky A. Choices, Values, and Frames. Am Psychol. 1984;39(4):341–350. 10.1037/0003-066X.39.4.341

[24] Dell’Ariccia G, Igan D, Laeven L. Credit booms and lending standard: Evidence from the subprime mortgage market. J Money Credit Bank. 2012;44(2–3):367–384.

[25] Ioannidou V, Ongena S, Peydró JL. Monetary Policy, Risk-Taking, and Pricing: Evidence from a Quasi-Natural Experiment. Rev Financ. 2014;19(1):95–144. 10.1093/rof/rfu035

[26] Brunnermeier MK. Deciphering the Liquidity and Credit Crunch 2007–2008. J Econ Perspect. 2009;23(1):77–100. 10.1257/jep.23.1.7720052301

[27] Kiminori M. Credit traps and credit cycles. Am Econ Rev. 2007;97(1):503–516. 10.1257/aer.97.1.503

[28] Maddaloni A, Peydró JL. Bank risk-taking, securitization, supervision and low interest rates: Evidence from lending standards. Rev Financ Stud. 2011;24(6):2121–2165. 10.1093/rfs/hhr015

[29] Bostic RW, Lee KO. Mortgages, risk and homeownership among low- and moderate-income families. Am Econ Rev. 2008;98(2):310–314. 10.1257/aer.98.2.310

