# Supplementary material for: The Regime Shift Associated with the 2004–2008 US Housing Market Bubble
Source: PLoS One. 2016 Sep 1;11(9):e0162140. doi: 10.1371/journal.pone.0162140 (PMC5008684; doi:10.1371/journal.pone.0162140)
Supplement: S1 File — Supporting information pdf file containing additional information on data and methodology. This is inclusive of figures, tables and text. (PDF) [file pone.0162140.s001.pdf]

Supporting Information: The regime shift associated with the 2004-2008 US housing market bubble

James P.L. Tan[1,3] and Siew Ann Cheong[2,3]

[1]Interdisciplinary Graduate School, Nanyang Technological University, 50 Nanyang Avenue, Block S2-B3a-01, Singapore 639798, Republic of Singapore
[2]Division of Physics and Applied Physics, School of Physical and Mathematical Sciences, Nanyang Technological University, 21 Nanyang Link, Singapore 637371, Republic of Singapore
[3]Complexity Institute, Complexity Institute, Block 2 Innovation Centre, Level 2 Unit 245, 18 Nanyang Drive, Singapore 637723

S1 Data

The data for the three US housing market variables were downloaded from Zillow (http://www.zillow.com/research/data/), which is an online real estate database company in the US. Because of its popularity (Zillow claims that it has data on more than 110 million US homes), its data represents a considerable cross-section of the US housing market. The three variables that we downloaded from Zillow are the median sale price (transaction price, Jul 97 - Dec 12), homes sold (transaction volume, Jul 97 - Dec 12), and % of homes sold for gain (Jan 98 - Feb 13). The data for these variables come in the form of monthly time series for a number of cities in the US. When computing the average normalized trajectory, in order for a comparison across variables, we only use data from time periods which the three variables overlap i.e. Jan 98 - Dec 12. It should be noted that we downloaded the data in early 2013. Due to an update by Zillow to the methodology of computing some variables in 2014, some of the data we used might not be available on the website now. In this case, requests for data should be directed either to Zillow or the corresponding author using the e-mail provided on the main text.

S2 Early Warning Signals

We calculate the lag-1 autocorrelation, standard deviation, spectral reddening, and skewness for the homes sold variable in overlapping 24-month time windows that we slide one month at a time. A time window length of 24 months was chosen as a balance between sensitivity to events in the housing market and resolution of the statistical early warning signals. The detection of statistically significant trends in aggregated early warning signals is robust to the choice of time window length (Section S8). Only cities that have no missing data from the homes sold variable are used to calculate these early warning signals.

From the theory of nonlinear dynamical systems, decreasing stability in a stable attractor means that recovery from perturbations take longer, giving rise to an increasing autocorrelation, spectral reddening of the power spectrum and an increasing standard deviation [8, 30]. Also, as the attractor becomes less stable, the energy well associated with it may become asymmetric [17]. In particular, the skewness also gives the direction in which the free energy is flattening. Let $\{r_t\}$, with indices indicating the time index, represent a detrended time series window for the transaction volume variable of the normalized average trajectory for any particular city. Then the lag-1 autocorrelation is calculated by the formula $R = E[(r_t - \mu)(r_{t+1} - \mu)]$, where μ is the mean and $\sigma^2 = E[(r_t - \mu)^2]/23$ is the variance. Before calculating the standard deviation of a time window, the parent time series of that time window is normalized by dividing by the standard deviation of the parent time series. Spectral reddening is quantified by calculating the median frequency $\omega_{1/2}$ of the frequency spectrum; a lower $\omega_{1/2}$ indicates a frequency spectrum that is more redshifted, having more power in the lower frequencies. The frequency spectrum is computed using a discrete Fourier transform (DFT), with the constant discarded and the frequency spectrum normalized. Finally, the skewness is computed according to the formula $E[(r_t - \mu)^3/\sigma^3]$.

Early warning signals for each time window were calculated. An early warning signal at any particular time was aggregated across all cities by counting the number of cities that pass a certain threshold for that early warning signal. This results in an aggregated early warning signal for the US housing market. The threshold was chosen such that the aggregated early warning signal was sensitive enough to the occurrence of statistically significant trends without

the threshold being too stringent such that the trends were undetectable (Figure A). The thresholds for the autocorrelation, standard deviation, spectral reddening and skewness are 0.5, 1.5, the third lowest frequency and 1.35 respectively.

Before calculating the statistical early warning signals, we detrend each time series using LOESS smoothing, after which individual time windows can be segmented from the time series of residues. This was done using the 'smooth' function in MATLAB with the 'rloess' method and a span of 40%. A span of 40% was chosen so that the smoothing fitted the slow dynamics well enough without overfitting the fast dynamics of the time series (Figure B). The detection of statistically significant aggregated early warning signals around the period of the two transitions is robust to the choice of span (Section S8).

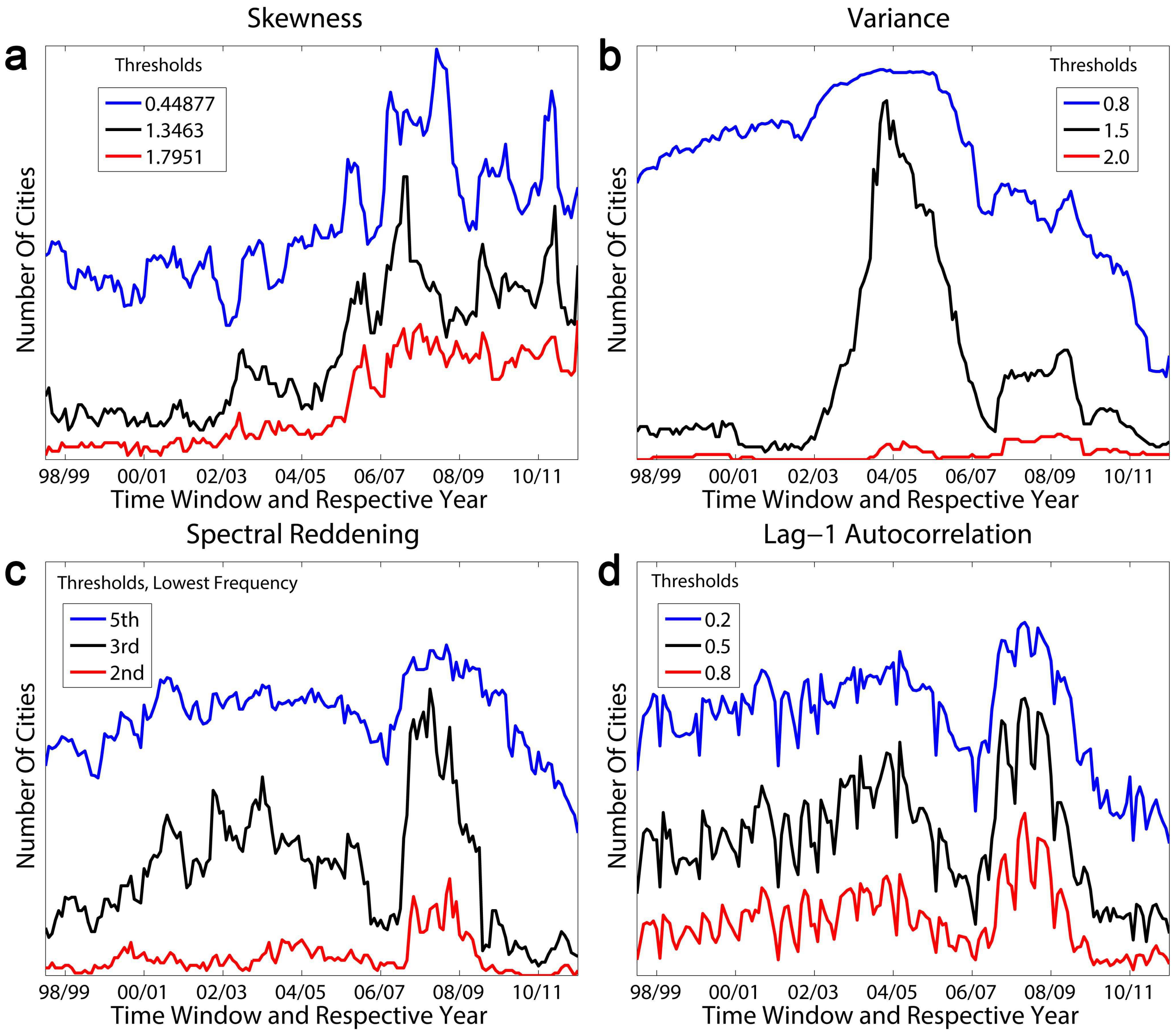

Figure A: Thresholds for early warning signals. Aggregated early warning signals for various thresholds are plotted to show the choice of thresholds used (the black plots). The plots are scaled by an appropriate factor to fit into the figure. Too loose or strict a threshold might conceal statistically significant early warning signals. Hence, the threshold should be chosen such that any statistically significant early warning signals may be detected.

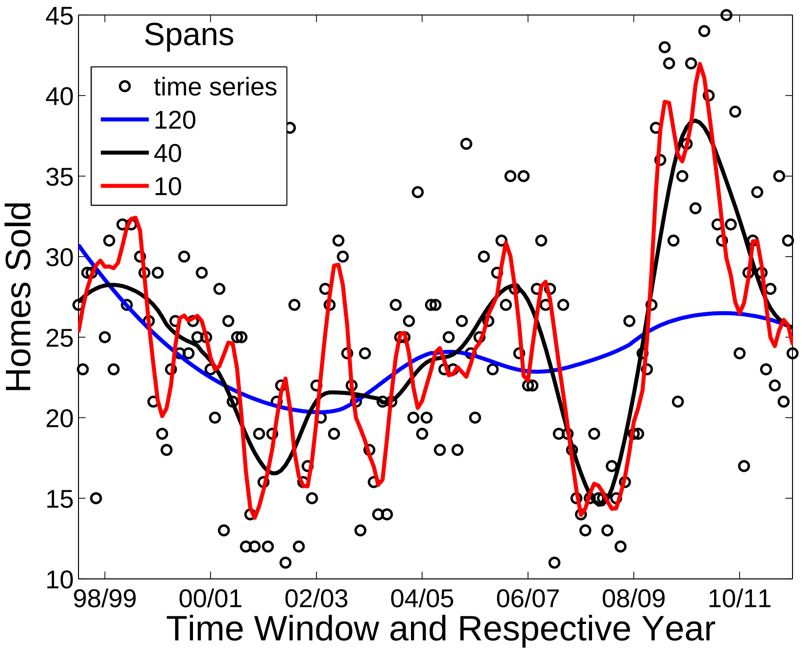

Figure B: Smoothing time series with different spans. Loess smoothing for different spans is shown for the homes sold time series of a city. Spans are given in terms of the percentage of data points in the time series. Too small a span (10%) overfits the data whereas too large a span (120%) averages out the slow dynamics. Sensitivity analysis of the statistical significance of trends of aggregated early warning signals with respect to span can be found in Section S8.

Significance Testing Significance testing of early warning signals is done by calculating the significance of the Kendall's τ [30], a measure of rank correlation between two random variables.

$$\tau = \frac{(\#\text{ of concordant pairs}) - (\#\text{ of disconcordant pairs})}{\frac{1}{2}n(n-1)} \tag{A}$$

In this case, one of the random variables is an aggregated early warning signal while the other is time. Hence, Kendall's τ measures the persistence of an increasing trend in early warning signal as the stability of the attractor decreases towards a phase transition point. The null model time series is created by bootstrapping from the time series of each city before the start of an increasing trend of aggregated early warning signal. For the spectral reddening and autocorrelation early warning signals, moving block bootstraps were used instead to retain the memory from the original time series [31]. We then calculated the early warning signal for the null model time series of each city and obtained the aggregated early warning signal which is the number of cities that passed a certain early warning threshold as described previously. After creating 10,000 such instances of aggregated early warning signal from null model time series, we can calculate the probability of obtaining a Kendall τ greater than or equal to that observed in the increasing trend. We consider the trend to be significant when the probability of obtaining such a Kendall τ due to random chance (null model) is less than 0.05. In Section S8, we conduct a sensitivity analysis on the span and the time window length and show that the detection of statistically significant aggregated early warning signals around the time of both transitions is robust.

Table A: Table of p-values for the statistical significance of kendall-τ observed in aggregated statistical early warning signals for the homes sold variable. The p-values are calculated for any statistically significant trend around the period of each transition and before the date assigned to each transition i.e. Oct 2003 for the Subprime Loans Transition and Sep 2008 for the Financial Crisis Transition. N/A indicates a trend that is either not statistically significant or has a negative kendall-τ.

	Skewness	Standard Deviation	Spectral Reddening	Autocorrelation
Subprime Loans Transition	0.0068	<0.0001	0.0018	0.0255
Subprime Loans Transition (signal before Oct 2003)	N/A	N/A	0.0018	N/A
Financial Crisis Transition	0.0042	0.0288	0.0004	0.0002
Financial Crisis Transition (signal before Sep 2008)	0.0042	N/A	0.0188	0.0001

S3 Average Normalized Trajectory

To study the trajectory of the US housing market in the phase space of the three variables, we partitioned each variable's time series into two-year sliding time windows for the purpose of a later comparison of the trajectory with the early warning signals. Each time window is normalized by dividing by the average of the entire time series, of which the time window is a constituent. The average normalized trajectory of the housing market for a variable at the time index of the time window is then calculated by averaging across the cities, the average of each city's time window for that variable. Doing this across all three variables and all time windows produces a time series which we used as the average normalized trajectory of the US housing market. Only cities that have no missing data from all three variables are used to calculate the average normalized trajectory.

S4 Fit Results of the Homes Sold Trajectory

The dynamical equation used in the main text is

$$\frac{\mathrm{d}x}{\mathrm{d}t} = -\frac{\partial U(x,t)}{\partial x}, \tag{B}$$

where $U(x,t)$ is the free energy approximated by a Taylor expansion

$$U(x,t) = -A(t)x + B(t)x^2 + C(t)x^3 + D(t)x^4. \tag{C}$$

Before fitting, it is possible to remove the third-order order term C_1 by a fixed translation in x so that $C_1 = 0$. Hence, $C(t)$ may be set to zero after the driving phase. We relate the homes sold trajectory $Q(t)$ to $x(t)$ by

$$Q(t) = \frac{\mathrm{d}x}{\mathrm{d}t} + Q_0 + Q_g t + Q_\Delta(t), \tag{D}$$

where

$$Q_\Delta(t) = \begin{cases} 0, & \text{for } t < t_\Delta; \\ \frac{Q_{\mathrm{end}} - Q_0}{t_f - t_\Delta}(t - t_\Delta), & \text{for } t_\Delta \le t \le t_f, \end{cases} \tag{E}$$

Table B: Table of fitting parameters and their 95% confidence interval in parentheses. The numerical value of t_Δ is the time window index, and corresponds to the Apr 05/Mar 07 time window.

	A_1	B_1	D_1	Q_{end}	x_1	t_Δ
Fitted values	0.29	-0.0056	3.1E-4	0.77	-0.22	73
95 % C.I.	(0.26, 0.32)	(-0.014, 0.0023)	(8.9E-5, 5.3E-4)	(0.76, 0.78)	(-2.1, 1.7)	(71, 75)

Determining Q_g, the natural growth rate Because US population growth was roughly linear for the past two decades, we assumed that the natural growth in $Q(t)$ is linear in time i.e. $Q_g t$ (Eq (D)). To estimate Q_g and Q_0, we perform a linear regression on $Q(9 \leq t \leq t_0)$ which is the linear portion of $Q(t)$ before the kink at t_0 when the driving was started. Here t is given in index units so $t = 9$ is the 9th time window in sequence. The linear regression gives the estimate $Q_g = 5.564E - 4\text{month}^{-1}$, with 95% confidence interval $(3.654E - 4, 7.473E - 4)$ and the estimate $Q_0 = 0.9782$ with the 95% confidence interval $(0.9772, 0.9792)$. The regression and plot of residuals can be found in Figure C.

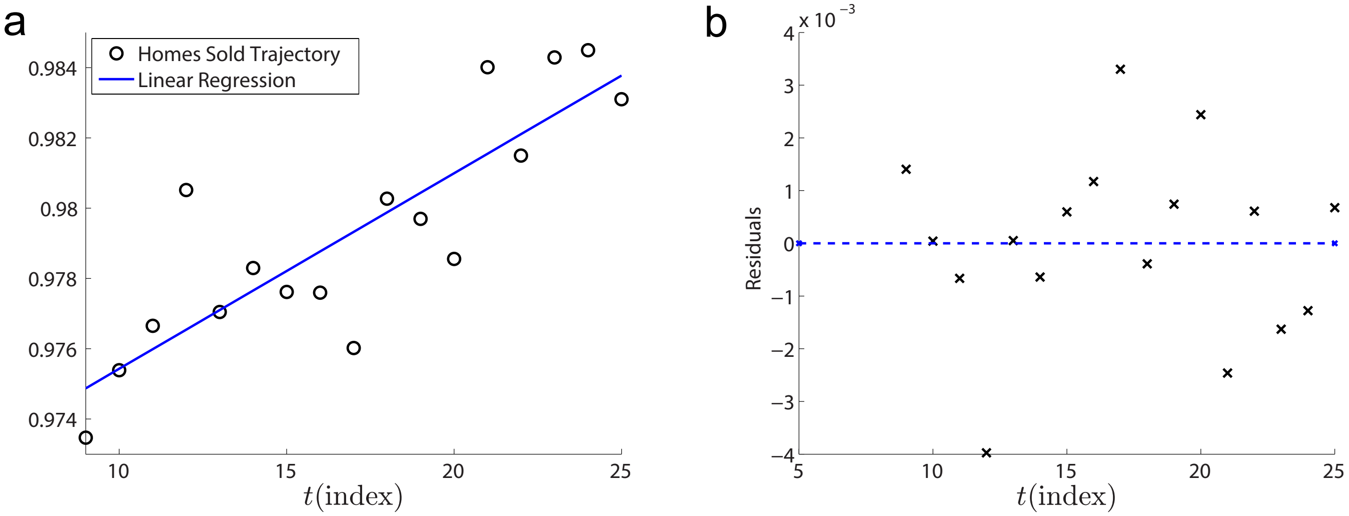

Figure C: A linear regression to estimate Q_g. (a) Linear regression of the homes sold trajectory preceding t_0. The linear regression is conducted for the purposes of estimating Q_g, the natural growth rate. (b) Plot of residuals.

Fitting algorithm All fits were done with MATLAB's lsqnonlin function, a nonlinear regression routine which uses a trust-region algorithm. Numerical integration of Eq (B) was done using a fourth-order Runge-Kutta method with a step size of 10^{-4} month. The parameters fitted were x_1, A_1, B_1, D_1, Q_{end}, and t_Δ, where x_1 is the initial condition.

Sensitivity analysis To ensure our model is robust, we explored alternatives to the assumptions used in the model, namely, concave and convex functions for $Q_\Delta(t)$ instead of the linear ones used in the model. We also accounted for different values of Q_g within the confidence interval estimated earlier. After fitting, our results Section S7 show that any fits with an R^2 larger than that of the original fit did not show any significant deviation of t_Δ that would affect the validity of the model used.

Endogeneity We used a linear regression for the time series preceding the transition so as to obtain a natural growth rate for the transaction volume in the United States. We assumed that this growth is linear because the population growth in the United States was roughly linear for the past few decades. In the Supporting Information Figure S3, we have plotted a regression and also a plot of residuals. Judging from the plot of residuals, we feel that the linear regression is satisfactory. Because we did sensitivity analysis for the parameters estimated from the linear regression, we feel that the model is robust even if there exists some explanatory variables that were omitted from the regression. For the regime shift model, it is safe to assume that not all endogenous variables are accounted for. However, since a large amount of variation in the homes sold variable can be explained by the model, we think that it is safer not to include more variables and run the risk of overfitting the data with variables that might only be spuriously related to the homes sold variable.

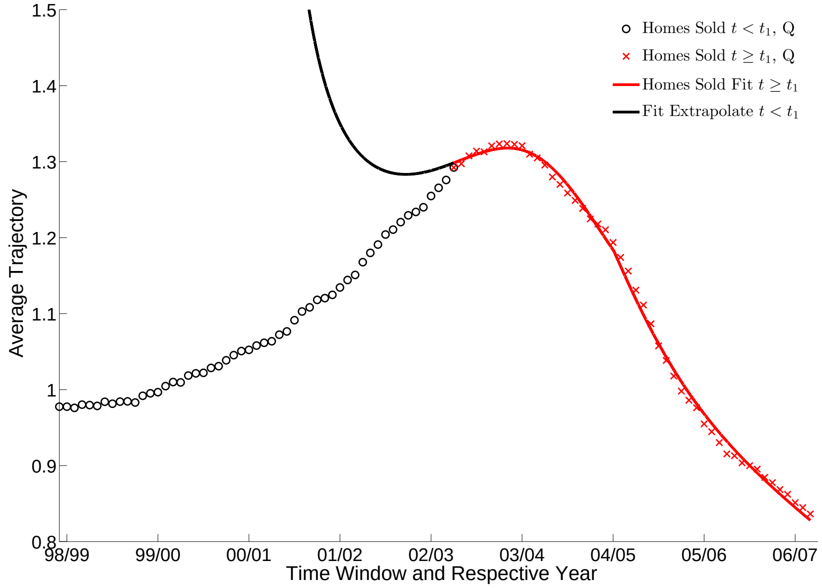

Figure D: Extrapolating from the fitted segment under a time reversal. The fitted parameters from the fit (red curve) indicate that only a stable fixed point exists after the driving phase. This rules out the possibility that $Q(t)$ may be explained by an absence of driving in the model. The black curve shows an extrapolation of the fitted dynamics under a time reversal from the red curve. The local minimum in the black curve shows a little ghosting, a remnant of the initial fixed point that was annihilated by the saddle node bifurcation during the driving phase, slowing trajectories moving through the region of phase space near the former position of the initial fixed point.

S5 Fitting $U(x, t)$ to simulated data of a grazing model

A commonly used one-dimensional grazing model in ecology exhibiting a regime shift is given by the dimensionless dynamical equation [32, 33]

$$\frac{dX}{d\tau} = X(1 - X) - \frac{\gamma X^2}{\alpha^2 + X^2}. \tag{F}$$

Here, X is the vegetation biomass as a proportion of its carrying capacity, γ controls the grazing rate, α determines at what level of X the grazing rate saturates and τ is the dimensionless time.

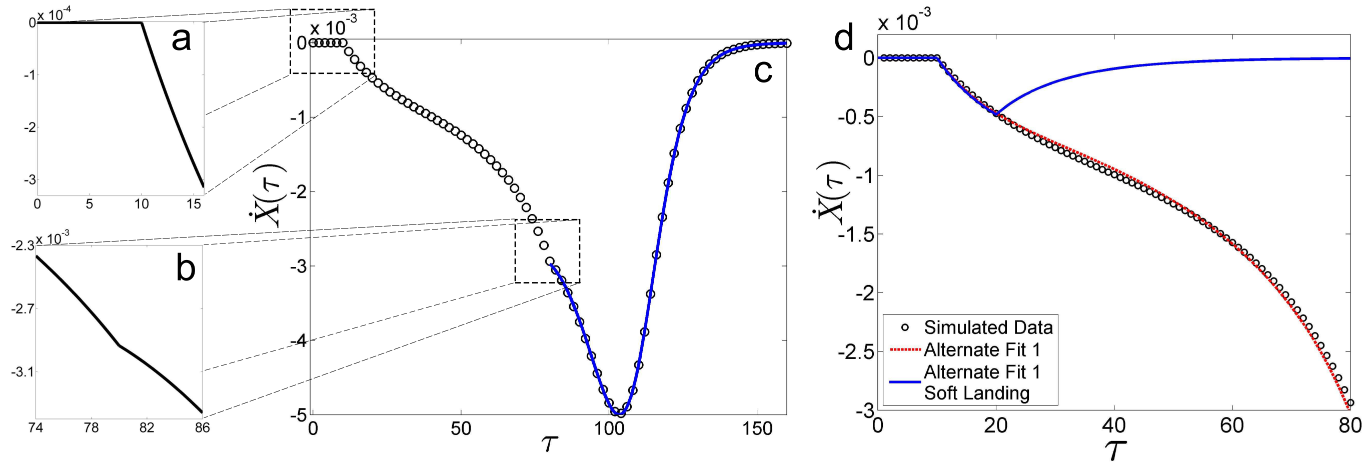

Figure E: Fitting Eq B to a grazing model with alternate stable states. The data points in (c) represent the simulated data from the modified grazing model. Here, $X(\tau)$ is the vegetation biomass as a proportion of the carrying capacity of the system while τ is dimensionless time. The blue line plot shows the fit of the approximated model (Eq B and C) to the data points after the driving force is stopped after $\tau = 80$. (a) and (b) are cut-outs of the simulated data, represented as line plots, which are centered on the kinks observed at $\tau = 10$ and $\tau = 80$ respectively. These kinks are due to the introduction and extinction of the driving force. Recovered parameters from the fit and details of the model used can be found in the Supporting Information. (d) Using Alternate Fit 1 of the modified grazing model (Table D) as an example, we demonstrate a soft landing without a phase transition by stopping the driving before the bifurcation occurs.

The model exhibits two stable fixed points when $\gamma = 0.23$ and $\alpha = 0.1$. Increasing γ causes a saddle-node bifurcation at $\gamma = 0.26$ which eliminates the stable fixed point with the larger vegetation biomass. If the system is initially at the stable fixed point with the larger vegetation biomass, the system will undergo a regime shift to the remaining stable fixed point which is at the smaller vegetation biomass.

We generate simulated data for $X(\tau)$ by numerically integrating Eq F. α is kept at a value of 0.1. From $0 \leq \tau \leq 10$, the system is initially at the high biomass stable fixed point. When $10 < \tau \leq 40$, $\gamma(\tau)$ is increased linearly with τ from $\gamma(10) = 0.23$ to $\gamma(40) = 0.28$. Then for $40 < \tau \leq 80$, γ is kept constant at 0.28. The simulated data $X(40 < \tau \leq 80)$ was then fitted to Eq B with free energy given by Eq C. Here, $A(40 < \tau \leq 80) = A_1$, $B(40 < \tau \leq 80) = B_1$, $C(40 < \tau \leq 80) = C_1 = 0$ and $D(40 < \tau \leq 80) = D_1$ are kept constant for the fit. The fitted parameters are A_1, B_1, D_1 and x_1, where x_1 is the initial position of the system at $\tau = 40$.

The approximation of Eq B to the grazing model given by Eq F depends on how close the system is to the target regime. We modified the grazing model to improve this approximation. This was done by adding a new parameter k to the grazing model resulting in the modified model

$$\frac{dX}{d\tau} = X(k - X) - \frac{\gamma X^2}{\alpha^2 + X^2}. \tag{G}$$

The parameters used in the modified model are $k = 0.9$ and $\alpha = 0.16$. From $0 \leq \tau \leq 10$, the system is initially at the high biomass stable fixed point. When $10 < \tau \leq 80$, $\gamma(\tau)$ is increased linearly with τ from $\gamma(10) = 0.231$ to $\gamma(80) = 0.236$. Then for $80 < \tau \leq 160$, $\gamma(\tau)$ is kept constant at 0.236. This modification allows us to decrease the distance between the initial regime and the final regime without substantially increasing the coefficients of the 5th order

and higher terms in Eq C thus improving the approximation. The results of the fit to simulated data from the original and modified model are shown in Table C. In Figure Ed, we contrast the behavior of the grazing model under extended forcing (resulting in biomass collapse), and when forcing is terminated early (allowing the biomass to achieve a soft landing).

Table C: Table of parameters generating simulated data from the grazing model and Eq B's fit to the simulated data. Values in brackets indicate the 95% confidence interval for the corresponding fitted parameters.

	Simulated	Fit	Simulated (modified)	Fit (modified)
A_1	-0.0486	-0.04406 (-0.045, -0.043)	-0.003896	-0.003942 (-0.00395, -0.00393)
B_1	-0.12	-0.144 (-0.15, -0.14)	-0.01449	-0.01497 (-0.0150, -0.0149)
D_1	0.977	1.754 (1.6, 1.9)	0.6832	0.8896 (0.882, 0.897)
x_1	0.1597	0.1334 (0.13, 0.14)	0.03791	0.03987 (0.0395, 0.0403)

S6 Overfitting the Driving Phase

In the main text, we alluded to the problem of overfitting the driving phase when estimating the functional forms of $A(\tau)$, $B(\tau)$, $C(\tau)$ and $D(\tau)$ without additional mechanistic insight. The problem lies in the large number of parameters involved relative to the simplicity of the curve being fitted. Additionally, error propagation from the estimation of A_1, B_1 and D_1 after the driving phase is also a problem when trying to model the driving phase. As an example, consider the modified grazing model specified in Section S5 and Eq G. We first try to estimate $A(\tau)$, $B(\tau)$, $C(\tau)$ and $D(\tau)$ during the driving phase ($10 < \tau \leq 80$) by assuming that $A(\tau)$, $B(\tau)$, $C(\tau)$ and $D(\tau)$ are linear functions,

$$Y(\tau) = \begin{cases} Y_0, & \text{for } 0 \leq \tau \leq 10, \\ Y_0, + \frac{Y_1 - Y_0}{70}(\tau - 10) & \text{for } 10 < \tau \leq 80, \\ Y_1, & \text{for } 80 < \tau \leq 160, \end{cases} \tag{H}$$

where Y represents A, B, C or D. In fact, $A(\tau)$, $B(\tau)$, $C(\tau)$ and $D(\tau)$ are indeed linear functions during the driving phase in this example because $\gamma(\tau)$ is driven linearly when generating the simulated data. We fit the driving phase of the simulated data to Eqs B, C and H after obtaining estimates of A_1, B_1 and D_1 from fitting the portion of data after the driving phase (Table C). The parameters to be fitted are thus A_0, B_0, C_0 and D_0. As we can see, very different parameters (Table D) fit the data almost equally well (Fig. F), and therefore we have a problem of the model overfitting the data.

S7 Sensitivity Analysis (Fitted Model)

The 95% confidence interval in the estimation of Q_g =5.564E-4 is (3.654E-4, 7.743E-4). We conduct the sensitivity analysis with five different values of Q_g: 3.654E-4, 4.609E-4, 5.564E-4, 6.518E-4 and 7.743E-4. We also explore different convex and concave functions for $Q_\Delta(t)$. For the convex functions, we use an exponential function of the form

$$Q_\Delta(t) = \frac{Q_{\text{end}} - Q_0}{\exp(\lambda t_f) - \exp(\lambda t_\Delta)}[\exp(\lambda t) - \exp(\lambda t_\Delta)] \tag{I}$$

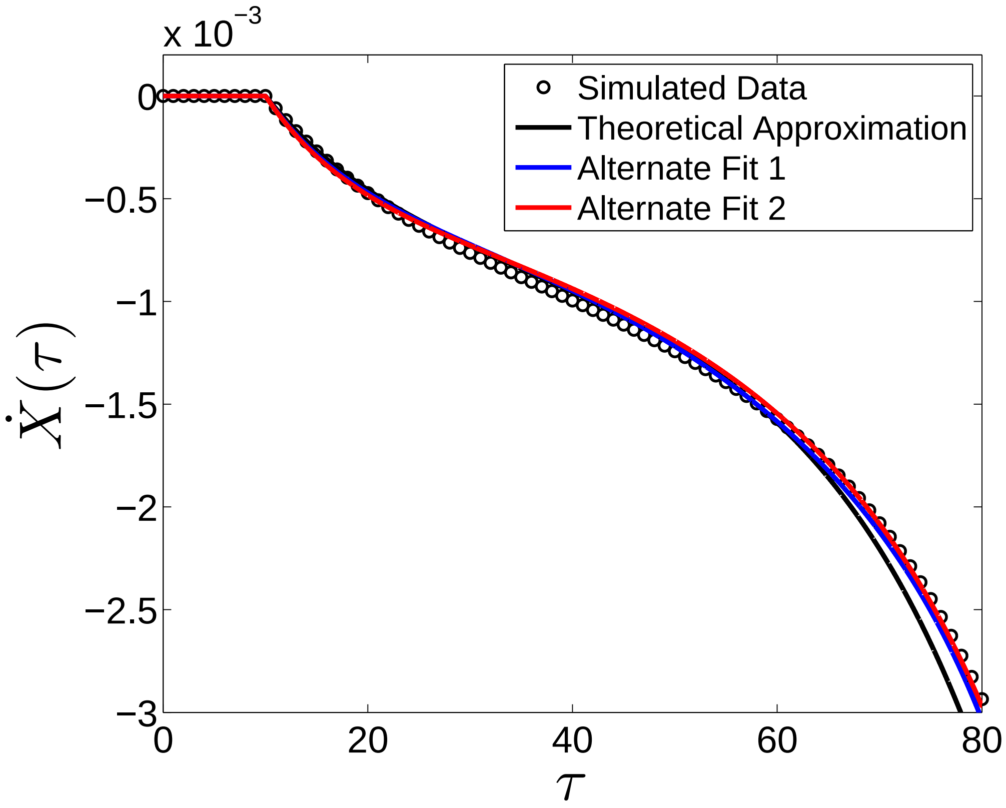

Figure F: Overfitting the driving phase. A comparison of the exact theoretical approximation (Eq B and C) with parameters calculated directly from the modified grazing model (Eq G) against two alternate local solutions obtained by fitting the approximation (Eq B and C) and the assumed linear driving forces (Eq H) to simulated data from the modified grazing model during the driving phase. A_1, B_1 and C_1 were calculated from the modified grazing model for all three plots. The fits are both better than the theoretical approximation as far as R^2 is concerned, but their parameters are very different, and also very different from the theoretical approximation (Table D).

Table D: Parameters recovered from two alternate local solutions to the fitting algorithm in addition to the R^2 and τ_b, which is the time the bifurcation occurred during the driving phase.

	A_0	B_0	C_0	D_0	τ_b	R^2
Theoretical Approximation	-3.6E-5	-0.017	-0.021	0.67	33.8	0.987
Alternate Fit 1	0.0029	0.042	1.6	2.4	36.6	0.998
Alternate Fit 2	7.0E-4	-0.0015	0.63	1.5	39.2	0.998

and a power function of the form

$$Q_\Delta(t) = \frac{Q_{\text{end}} - Q_0}{(t_f - t_\Delta)^\lambda}(t - t_\Delta)^\lambda. \tag{J}$$

For the convex exponential function, we fit for values of λ starting from 5E-3, increasing in increments of 5E-3 until 7E-2. For the convex power function, we fit for values of λ starting from 1.05, increasing in increments of 0.05 until 1.70. For the concave functions, we use the power function given by Eq J and the logarithmic function found by inverting Eq I and applying the appropriate linear transformations. For the concave power function, we fit for values of λ

starting from 0.3, increasing in increments of 0.05 to 0.95. For the logarithmic function, we fit for values of λ starting from 5E-3, increasing in increments of 5E-3 until 7E-2. Hence, increasing λ is associated with an increasing curvature in $Q_\Delta(t)$ for the exponential, logarithmic and convex power function whereas increasing λ is associated with a decreasing curvature in $Q_\Delta(t)$ for the concave power function. The results of the sensitivity analysis may be found in Figure G. The distribution of t_Δ and its 95% confidence interval bounds for fits with an R^2 greater than the original fit can be found in Figure H. The results of the sensitivity analysis show that the estimation of t_Δ is robust for the alternative scenarios considered in the sensitivity analysis.

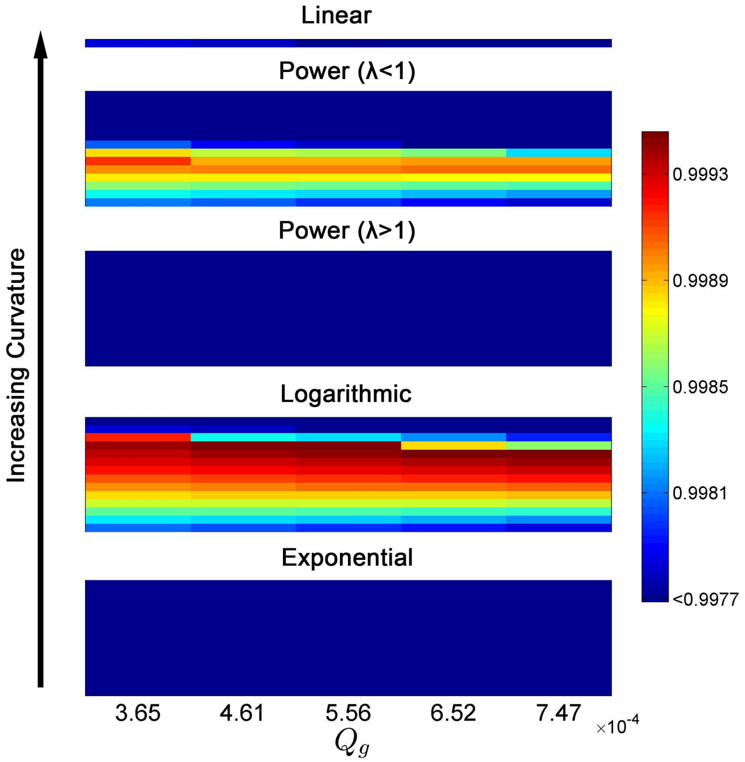

Figure G: R^2 values in the parameter space. A heat map plot of the R^2 values (in color) obtained from fits of various values of Q_g and functions of $Q_\Delta(t)$ with various values of λ. The vertical axis shows the various functions tested for $Q_\Delta(t)$, and λ within each function along the vertical axis is sorted according to increasing curvature of $Q_\Delta(t)$. Any R^2 below 0.9978, which is the R^2 of the original fit, is relegated to a single color.

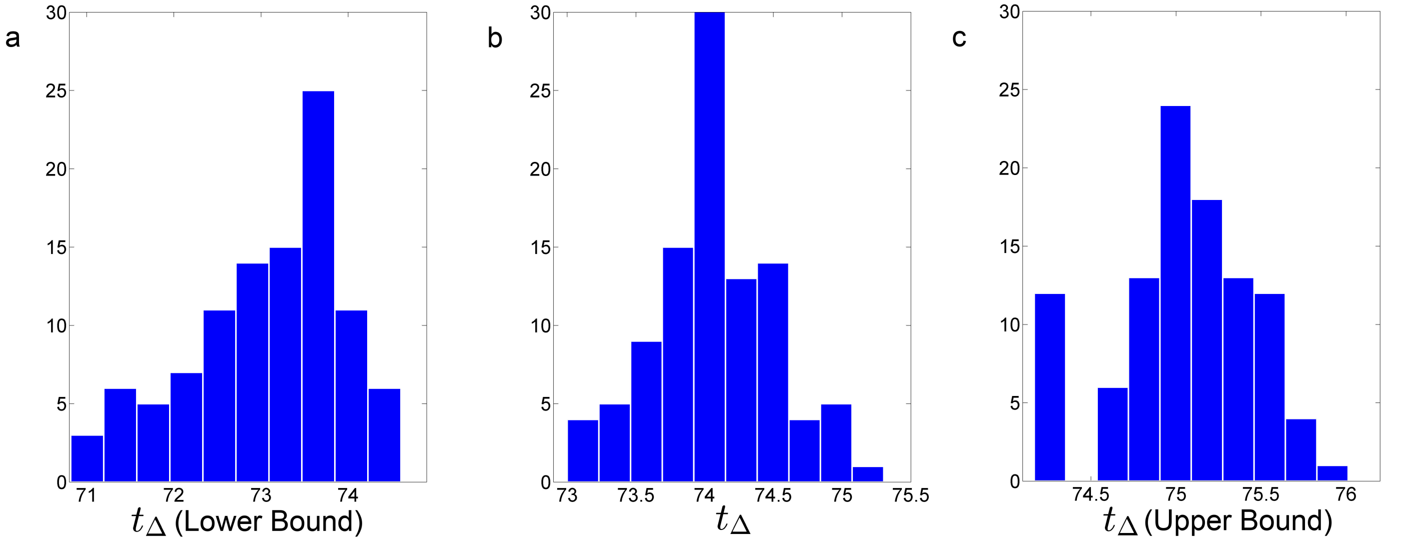

Figure H: Distributions of fitted t_Δ from the sensitivity analysis. Histograms of t_Δ from fits of Figure G with an R^2 greater than the original fit. (a) The lower bound of the 95% confidence interval of t_Δ. (b) t_Δ. (c) The upper bound of the 95% confidence interval of t_Δ.

S8 Sensitivity Analysis (Early Warning Signals)

The following tables contain the sensitivity analysis of the p-values of increasing trends in aggregated early warning signals around the Subprime Loans Transition and the Financial Crisis Transition with respect to the span and time window lengths. The span is given as a percentage of the total number of data points in the time series. 1,000 samples were obtained to compute each p value except for the parameters used for presenting the results of the main text where the span is 40% and the time window length is 24. 10,000 samples were used in this case.

Table E: Table of p-values for the significance of aggregated lag-1 autocorrelation signals around both transitions calculated for various span percentages and time window lengths. Shaded cells indicate p-values above the 5% significance level. The argument of $p(\cdot)$ is the number of months in a time window.

**span(%)**	p**(12)**	p**(18)**	p**(24)**	p**(30)**	p**(36)**
Subprime Loans Transition					
20	0.004	0.001	0.004	0.001	0.001
30	<0.001	0.011	0.015	0.004	0.019
40	<0.001	0.035	0.0255	0.011	0.024
50	<0.001	0.003	0.012	0.007	0.01
60	<0.001	0.004	0.01	0.013	0.013
70	<0.001	0.001	0.013	0.02	0.014
80	<0.001	0	0.014	0.02	0.003
Financial Crisis Transition					
20	0.063	0.027	0.007	0.106	0.021
30	0.002	0.008	0.002	0.003	0.011
40	<0.001	<0.001	0.0002	<0.001	0.002
50	<0.001	<0.001	<0.001	<0.001	<0.001
60	<0.001	<0.001	<0.001	<0.001	0.005
70	<0.001	<0.001	<0.001	0.001	0.003
80	<0.001	<0.001	<0.001	0.001	0.001

Table F: Table of p-values for the significance of aggregated spectral reddening signals around both transitions calculated for various span percentages and time window lengths. Shaded cells indicate p-values above the 5% significance level. The argument of $p(\cdot)$ is the number of months in a time window.

**span(%)**	p**(12)**	p**(18)**	p**(24)**	p**(30)**	p**(36)**
Subprime Loans Transition					
20	<0.001	0.014	0.027	<0.001	0.002
30	<0.001	0.023	0.005	0.022	<0.001
40	<0.001	0.028	0.0018	0.016	<0.001
50	0.001	0.006	0.004	0.02	<0.001
60	<0.001	0.012	0.002	0.001	<0.001
70	0.001	0.004	0.002	0.025	<0.001
80	<0.001	0.045	0.007	0.021	<0.001
Financial Crisis Transition					
20	0.014	0.031	0.114	0.002	<0.001
30	<0.001	0.009	<0.001	0.011	<0.001
40	<0.001	0.002	0.0004	0.001	<0.001
50	<0.001	0.002	0.002	0.001	<0.001
60	<0.001	0.005	0.005	0.001	<0.001
70	<0.001	0.007	0.001	<0.001	<0.001
80	<0.001	0.023	0.001	0.001	<0.001

Table G: Table of p-values for the significance of aggregated skewness signals around both transitions calculated for various span percentages and time window lengths. Shaded cells indicate p-values above the 5% significance level. The argument of $p(\cdot)$ is the number of months in a time window.

**span(%)**	p**(12)**	p**(18)**	p**(24)**	p**(30)**	p**(36)**
Subprime Loans Transition					
20	0.054	0.052	0.001	0.006	0.001
30	0.009	0.001	0.001	$<$0.001	0.017
40	0.007	0.004	0.0068	0.001	0.034
50	0.019	0.003	0.017	0.005	0.033
60	0.005	0.002	0.03	0.008	0.042
70	0.026	0.009	0.038	0.002	0.004
80	0.023	0.006	0.039	0.007	0.012
Financial Crisis Transition					
20	0.004	0.004	0.009	$<$0.001	$<$0.001
30	0.039	0.003	0.008	0.004	$<$0.001
40	0.067	0.002	0.0042	$<$0.001	$<$0.001
50	0.037	0.002	0.003	0.001	$<$0.001
60	0.041	0.001	0.004	$<$0.001	$<$0.001
70	0.022	0.003	0.003	$<$0.001	$<$0.001
80	0.047	0.007	0.005	$<$0.001	$<$0.001

Table H: Table of p-values for the significance of aggregated standard deviation signals around both transitions calculated for various span percentages and time window lengths. Shaded cells indicate p-values above the 5% significance level. The argument of $p(\cdot)$ is the number of months in a time window.

**span(%)**	p**(12)**	p**(18)**	p**(24)**	p**(30)**	p**(36)**
Subprime Loans Transition					
20	0.026	<0.001	<0.001	<0.001	<0.001
30	0.006	<0.001	<0.001	<0.001	<0.001
40	<0.001	<0.001	<0.0001	<0.001	<0.001
50	<0.001	<0.001	<0.001	<0.001	<0.001
60	<0.001	<0.001	<0.001	<0.001	<0.001
70	<0.001	<0.001	<0.001	<0.001	<0.001
80	<0.001	<0.001	<0.001	<0.001	<0.001
Financial Crisis Transition					
20	0.017	<0.001	<0.001	0.001	<0.001
30	<0.001	<0.001	0.017	<0.001	<0.001
40	0.007	0.001	0.0288	0.015	0.02
50	0.048	<0.001	0.002	0.022	0.022
60	<0.001	0.021	0.006	0.015	0.064
70	<0.001	0.033	<0.001	0.026	0.002
80	<0.001	0.041	<0.001	0.015	0.003

References

1. Benjamin J. Keys, Tanmoy Mukherjee, Amit Seru, and Vikrant Vig. Did securitization lead to lax screening? Evidence from subprime loans. *Q. J. Econ.*, 125(1):307–362, 2010.

2. Atif Mian and Amir Sufi. The consequences of mortgage credit expansion: Evidence from the U.S. mortgage default crisis. *Q. J. Econ.*, 124(4):1449–1496, 2009.

3. Yuliya Demyanyk and Otto Van Hemert. Understanding the subprime mortgage crisis. *Rev. Financ. Stud.*, 24(6):1848–1880, 2011.

4. Wei Xing Zhou and Didier Sornette. Is there a real-estate bubble in the US? *Physica A*, 361(1):297–308, 2006.

5. Takaaki Ohnishi, Takayuki Mizuno, Chihiro Shimizu, and Tsutomu Watanabe. Power laws in real estate prices during bubble periods. *Int. J. Mod. Phys. Conf. Ser.*, 16:61–81, 2012.

6. Hao Meng, Wen Jie Xie, Zhi Qiang Jiang, Boris Podobnik, Wei Xing Zhou, and H. Eugene Stanley. Systemic risk and spatiotemporal dynamics of the us housing market. *Sci. Rep.*, 4(3655):1–7, 2014.

7. James P. L. Tan and Siew Ann Cheong. Critical slowing down associated with regime shifts in the us housing market. *Eur. Phys. J. B*, 87(38):1–10, 2014.

8. Marten Scheffer, Jordi Bascompte, William A. Brock, Victor Brovkin, Stephen R. Carpenter, Vasilis Dakos, Hermann Held, Egbert H. van Nes, Max Rietkerk, and George Sugihara. Early-warning signals for critical transitions. *Nature*, 461(7260):53–59, 2009.

9. Annelies J. Veraart, Elisabeth J. Faassen, Vasilis Dakos, Egbert H. van Nes, Miquel Lü, and Marten Scheffer. Recovery rates reflect distance to a tipping point in a living system. *Nature*, 481(7381):357–359, 2012.

10. Chris A. Boulton, Lesley C. Allison, and Timothy M. Lenton. Early warning signals of atlantic meridional overturning circulation collapse in a fully coupled climate model. *Nat. Commun.*, 5(5752):1–9, 2014.

11. Martin Krkošek and John M. Drake. On signals of phase transitions in salmon population dynamics. *Proc. R. Soc. B*, 281(1784):20133221, 2014.

12. Ingrid A. van de Leemput, Marieke Wichers, Angelique O. J. Cramer, Denny Borsboom, Francis Tuerlinckx, Peter Kuppens, Egbert H. van Nes, Wolfgang Viechtbauer, Erik J. Giltay, Steven H. Aggen, Catherine Derom, Nele Jacobs, Kenneth S. Kendler, Han L. J. van der Maas, Michael C. Neale, Frenk Peeters, Evert Thiery, Peter Zachar, and Marten Scheffer. Critical slowing down as early warning for the onset and termination of depression. *Proc. Natl. Acad. Sci. USA*, 111(1):87–92, 2014.

13. Christian Meisel and Christian Kuehn. Scaling effects and spatio-temporal multilevel dynamics in epileptic seizures. *PLOS ONE*, 7(2):1–11, 2012.

14. S. R. Carpenter, J. J. Cole, M. L. Pace, R. Batt, W. A. Brock, T. Cline, J. Coloso, J. R. Hodgson, J. F. Kitchell, D. A. Seekell, L. Smith, and B. Weidel. Early warnings of regime shifts: A whole-ecosystem experiment. *Science*, 332(6033):1079–1082, 2011.

15. H. Held and T. Kleinen. Detection of climate system bifurcations by degenerate fingerprinting. *Geophys. Res. Lett.*, 31(23):L23207, 2004.

16. Rong Wang, John A. Dearing, and Peter G. Langdon. Flickering gives early warning signals of a critical transition to a eutrophic lake state. *Nature*, 492(7429):419–422, 2012.

17. V. Guttal and C. Jayaprakash. Changing skewness: An early signal of regime shifts in ecosystems. *Ecol. Lett.*, 11(5):450–460, 2008.

18. Y. L. Xu. Does mortgage deregulation increase foreclosures? Evidence from cleveland. *Reg. Sci. Urban Econ.*, 46:126–139, 2014.

19. Patricia A. McCoy, Andrey D. Pavlov, and Susan M. Wachter. Systemic risk through securitization: The result of deregulation and regulatory failure. *Conn. L. Rev.*, 41:493, 2009.

20. Dan Immergluck. Core of the crisis: Deregulation, the global savings glut and financial innovation in the subprime debacle. *City Community*, 8(3):341–345, 2009.

21. F.S. Mishkin. Over the cliff: From the subprime to the global financial crisis. *J. Econ. Perspect.*, 25(1):49–70, 2011.

22. L. Randall Wray. Lessons from the subprime meltdown. *Challenge*, 51(2):40–68, 2008.

23. D. Kahneman and A. Tversky. Choices, values, and frames. *Am. Psychol.*, 39(4):341–350, 1984.

24. G. Dell'Ariccia, D. Igan, and L. Laeven. Credit booms and lending standard: Evidence from the subprime mortgage market. *J. Money Credit Bank*, 44(2-3):367–384, 2012.

25. V. Ioannidou, S. Ongena, and J. L. Peydró. Monetary policy, risk-taking, and pricing: Evidence from a quasi-natural experiment. *Rev. Financ.*, 19(1):95–144, 2014.

26. M.K. Brunnermeier. Deciphering the liquidity and credit crunch 2007-2008. *J. Econ. Perspect.*, 23(1):77–100, 2009.

27. M. Kiminori. Credit traps and credit cycles. *Am. Econ. Rev.*, 97(1):503–516, 2007.

28. A. Maddaloni and J.L. Peydró. Bank risk-taking, securitization, supervision and low interest rates: Evidence from lending standards. *Rev. Financ. Stud.*, 24(6):2121–2165, 2011.

29. R.W. Bostic and K.O. Lee. Mortgages, risk and homeownership among low- and moderate-income families. *Am. Econ. Rev.*, 98(2):310–314, 2008.

30. Vasilis Dakos et al. Methods for detecting early warnings of critical transitions in time series illustrated using simulated ecological data. *PLOS ONE*, 7(7):1–20, 2012.

31. Hans R. Künsch. The jackknife and the bootstrap for general stationary observations. *The Annals of Statistics*, 17(3):1217–1241, 1989.

32. Robert M. May. Thresholds and breakpoints in ecosystems with a multiplicity of stable states. *Nature*, 269(6):471–477, 1977.

33. Imanuel Noy-Meir. Stability of grazing systems: An application of predator-prey graphs. *Journal of Ecology*, 63(2):459–481, 1975.
